# Hemorrhage of the Ramus Lumbalis of the Iliolumbar Artery as a Cause for Shock in Blunt Trauma Victims on Therapeutic Anticoagulation

**DOI:** 10.1155/2021/8870154

**Published:** 2021-01-15

**Authors:** Thomas Patrick Sullivan, Eduardo Smith-Singares

**Affiliations:** ^1^Memorial Hospital of Carbondale-The Barnes Jewish Collaborative, USA; ^2^Washington University School of Medicine in St. Louis, USA

## Abstract

Noncavitary torso hemorrhage is a rare and poorly characterized injury that can lead to exsanguination if not promptly addressed. When present in a high-risk patient on therapeutic anticoagulation, it can lead to a swift fatal outcome. Two cases (an 80-year-old female on warfarin and a 67-year-old male on apixaban for atrial fibrillation) presented with shock after direct blunt trauma in their torsos. Embolotherapy techniques were utilized to obtain angiostasis while the patients were resuscitated with massive transfusion protocols and reversal of the agents received. In the setting of severe localized blunt trauma on an aging victim while on antithrombotic medications, noncavitary torso hemorrhage must be included in the differential diagnosis. Local expertise and a high level of suspicion were critical in the early intervention, and postprocedural management of the injuries sustained and secured a good result.

## 1. Introduction

Uncontrolled bleeding is the most common cause of preventable death in trauma victims [1]. Multiple circumstances play a role in the timely arrest of hemorrhage (and ultimately in survival), including transport times [2], expeditious evaluation by an experienced trauma team, and the availability of resources to obtain hemostasis, surgically or otherwise [3]. The state of Illinois has a robust trauma system (established in 1971 as the first in the United States), and it conducted a feasibility study (published in 2015) [4] which ultimately resulted in the foundation of the Trauma Center at Memorial Hospital of Carbondale, a Barnes-Jewish Collaborative member located in the last designated trauma desert in the state [4]. New protocols have been initiated for the management of traumatic injuries including a two-tiered trauma response (see Table 1). These factors integrate to form a safety network that makes otherwise lethal injuries survivable. Their relevance only increases when difficult medical comorbidities complicate a rare presentation or an unusual injury that requires both a high index of suspicion and targeted therapy, while avoiding potential pitfalls. The two cases below exemplify these dynamics in our newly designated trauma center serving an otherwise austere environment.

## 2. Case Presentation

### 2.1. First Case

A 67-year-old (yo) lumberjack arrived with a category I activation after being airlifted from the scene of a work accident. The prehospital report described the patient as found lying next to the trunk he just felled. His systemic arterial blood pressure (BP) was 60/30 during the flight, but his pulse was in the 30-40 range. Ringer's lactate (Baxter International, Deerfield, IL) was infused through an 18-gauge (Ga) intravenously (IV) in the arm, and his transport time was 21 minutes. Upon arrival, he was placed on the monitors and his clothes were removed. He was intermittently conscious and confused regarding the details of the incident. His initial vitals were 75/42 and 54 with a right bundle branch block pattern and diffuse T-wave inversion. He was arousable and oriented in person only. His respiratory sounds were equal bilaterally, and his heart tones appeared normal. He complained of chest pain and lower back pain. A Focused Assessment with Sonography in Trauma with added limited echocardiography (E-FAST) exam was negative for fluid, and both his abdominal palpation and pelvic stability were deemed normal. The massive transfusion protocol (MTP) was initiated as we got report from a family member: he had a history of coronary artery disease (2 stents 4 months before presentation) and atrial fibrillation (ablated 2 years prior) and was receiving apixaban (Eliquis, Bristol-Myers Squibb, New York, NY) and clopidogrel (Plavix, Bristol-Myers Squibb, New York, NY). Upon logrolling the patient, a firm, slightly bruised mass was found in his right paralumbar area, extending to his right lateral iliac crest, tender on palpation. After 2 units (U) of O-negative, 5 U of cross-matched packed red blood cells (PRBC), 5 U of fresh frozen plasma (FFP), two units of platelets, 1000 mg of tranexamic acid (TXA) (Cyklokapron, Pfizer, New York, NY), and 4500 mg of 4F-PCC (Kcentra, CSL Behring, King of Prussia, PA), his vitals were 85/48 and 62. Pelvic X-rays failed to show any bony fractures or diastasis symphysis pubis.

**Figure 1:**
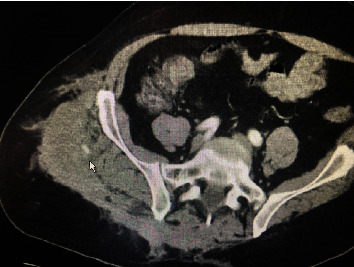
CT scan of the abdomen and pelvis for the first patient; the white arrow points towards a site of active extravasation (“blush”).

**Figure 2:**
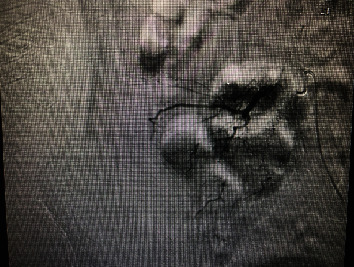
Digital subtraction angiography screenshot for the first patient; there is a visible pseudoaneurysm.

**Figure 3:**
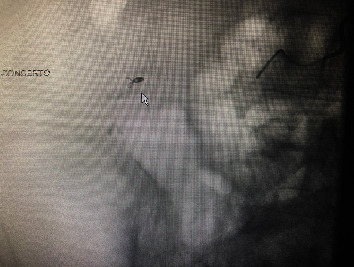
Digital subtraction angiography screenshot for the first patient after embolization was completed; the white arrow points towards the coiled blood vessel.

The patient was then transported to the CT scanner while still connected to the Belmont infuser (Belmont Medical, Billerica, MA) and receiving the rest of the first round of the MTP. The CT showed a very large hematoma within the confines of the abdominal wall, with a clear arterial phase extravasation (blush) (Figure 1, arrow). The only other injury noted in the study was a nondisplaced fracture of the right transverse process of L1. The head CT was read as within normal limits. He was then transported to the Interventional Radiology (IR) suite, where his left femoral artery was cannulated. Selective angiography examinations of his aorta and common iliacs, right hypogastric trunk, right iliolumbar branch, and L4 right lumbar branches were performed, revealing a pseudoaneurysm of the distal ramus lumbalis of the right iliolumbar artery cranial and medial to the right iliac crest (Figure 2), which was embolized using Gelfoam (Pfizer, New York, NY) slurry and Ruby coils (Penumbra, Alameda, CA), obtaining angiostasis (Figure 3, arrow). Additional embolotherapy was administered to the L4 right lumbar artery anastomosis to the deep right iliac circumflex artery. Postprocedure, the patient was transferred to the ICU, where his vitals were 185/99 and HR 78. Serial troponins rose modestly to 0.56, and cardiology recommended resuming clopidogrel upon discharge. The skin of his right lower back bruised up over the next 48 hours, but the mass remained stable. An additional follow-up was arranged for cardiology for the resumption of his apixaban as an outpatient.

### 2.2. Second Case

**Figure 4:**
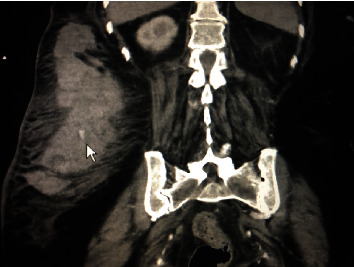
CT scan of the abdomen and pelvis for the second patient; the white arrow points towards a site of active extravasation (“blush”).

An 80 yo lady was brought in with a category II activation (transfer) after sustaining a fall from standing height at home. The patient stated that she lost her footing and hit a wooded armoire in the right side of her lower back. She was initially transported to a critical access hospital, where the only finding in her physical exam was a very large hematoma on the right side of her back, and she was mildly hypotensive (88/50). Her initial BP on arrival to the trauma center (after 2 L of Ringer's lactate) was 101/52. Her primary survey confirmed the findings at the other hospital. Her past medical history was significant for atrial fibrillation, hypertension, and diabetes. Her medications included a beta blocker, an oral hypoglycemic agent, low-dose acetylsalicylic acid (Ecotrin, Prestige Consumer Healthcare Inc., Greenburgh, NY), and warfarin (Coumadin, Bristol-Myers Squibb, New York, NY). STAT hemoglobin was measured as 7.2 g/dL (a drop from 14 two months prior). Her International Normalized Ratio (INR) was measured at 4.02. A CT scan of the abdomen and pelvis was obtained (Figure 4), showing an 18.3 cm × 4.9 cm × 16.8 cm right flank hematoma with active extravasation. Additional injuries included nondisplaced fractures of the right transverse processes of L1 and L2. Upon return to the trauma bay, her blood pressure dropped again to the high 80's. The Belmont infuser was connected, and 2 units of fresh frozen plasma with 2 units of packed red blood cells were rapidly infused. In addition, 10 mg of IV vitamin K was given, while the IR team prepared the angiography suite.

The patient was then transported to the IR suite, where her right femoral artery was cannulated.

**Figure 5:**
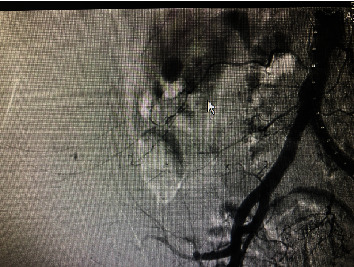
Digital subtraction angiography screenshot for the second patient; there is a visible pseudoaneurysm.

**Figure 6:**
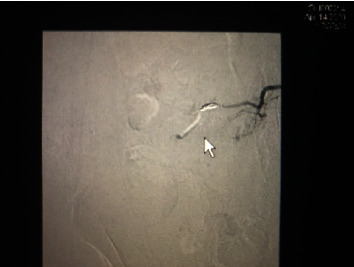
Digital subtraction angiography screenshot for the second patient after embolization was completed; the white arrow points towards the coiled blood vessel.

Selective angiography examinations of her aorta and common iliacs, right hypogastric trunk, right iliolumbar branch, and L4 right lumbar branches were performed, revealing active hemorrhage from an anastomotic vessel between the right L4 and the ramus lumbalis of the right iliolumbar artery (Figure 5). Gelfoam slurry followed by 2 Nester 2 × 4 mm microcoils (Cook Medical, Bloomington, IN) was embolized to stasis, confirmed by digital subtraction (Figure 6). The rest of the angiography was within normal limits. Postprocedure, she was transported to the ICU, where her vitals remained stable.

## 3. Outcomes and Follow-Up

### 3.1. First Case

He was discharged on hospital day 3. No complications were found at 60-day follow-up, and he resumed his commercial logging activities. With the onset of the COVID-19 emergency restrictions, he was furloughed. We contacted him at home by phone, and he has developed arthritic right knee pain. The hematoma has begun liquefying, and he will be returning to have it drained if it does not disappear by the time he is due back at the logging field.

### 3.2. Second Case

She was deemed to be too frail to return home immediately and was discharged to acute inpatient rehab 72 hours after the procedure, and she remained there for a week until she was discharged home. On her first follow-up (4 weeks after the injury), she remained in her usual state of health. Her hematoma had liquefied as well and was experiencing regression in size. She was readmitted 7 weeks after the injury with neurological symptoms suggestive of an ischemic stroke. Her lesion was diminished in size compared to her previous follow-up. She was considered to have contraindications for the use of TPA, and given her general medical condition, no neurointerventions were recommended. She was discharged to neurorehabilitation with continued aphasia.

## 4. Discussion

Traumatic shock carries severe morbidity and mortality that correlate with the time required to stop all bleeding [1, 2]. Understanding the mechanism of injury is critical to anticipate the most likely sources of hemorrhage and provide measures geared towards hemostasis. Extremity bleeding is easily identified, and control might be initiated at the scene with the use of splints to reduce fractures and tourniquets. Visible blood loss responds to direct compression and surgical interventions aimed at terminating exsanguination and restoring distal perfusion. Torso hemorrhages most commonly occur within the pleural or the peritoneal cavities, which are not compressible and require operative management. They require a high index of suspicion and a certain level of expertise to diagnose promptly and manage appropriately [3, 5]. Virtual spaces such as the retroperitoneum and the pelvic space are closely linked to specific lesions and mechanisms of injury (i.e., pelvic fractures, deceleration trauma, and seatbelts) [5].

Noncavitary torso bleeding is a very rare cause for hemorrhagic shock [6–9]. While the mortality potential for this noncompressible hematoma has been documented before [10, 11], the associated risk factors are poorly understood: a brief revision of the reported few cases in the available biomedical literature suggests some common features: advanced age [6–11] and localized high-energy blunt force in the lower back [6–9], with (or most frequently without) associated fractures in the lumbar spine and shearing of an arterial bleeder that dissects an otherwise closed space (either at the skin or in the fascial layers of the back or the buttocks) [6–9]. The most commonly associated blood vessel is the iliolumbar artery [9, 12], a surgically difficult-to-access vessel with significant anatomical variations [12–14], considered an inconsistent part of the corona mortis [15].

There have been a number of recent cases reported in the context of anticoagulation for atrial fibrillation and other indications that add a layer of complexity to the injury [16]. It is well known that patients that sustain trauma (of any kind) while receiving anticoagulation are older, have more comorbidities and higher injury severity scores, and have the worst outcomes compared to the general trauma population [3, 16]. Within the United States, they also cluster in more exurban areas, which are poorly served by specialist care [2]. It is known that properly planned trauma networks are effective at closing the gap of care between urban and rural trauma by bringing needed expertise closer to the injury site [1]. The two cases reported above show those dynamics at work: an elderly exsanguinating patient, with severe comorbidities and affected by a rare injury requiring significant expertise not usually available at austere settings and unlikely to survive through transfer to a remote regional trauma center, having a good outcome secondary to a high index of suspicion and immediately available resources. One issue that remains controversial is the most appropriate timing to resume anticoagulation (both antiplatelet agents and factor X inhibitors) as the management strategies and different final outcomes above illustrate. A review of the literature [17] shows different windows for the most common agents, depending upon their putative mechanism of action and the type of procedure received.

## 5. Conclusion

Noncavitary torso bleeding is a rare and potentially lethal form of noncompressible traumatic injury, which seems to affect older patients with more severe comorbidities that sustain localized, high-energy blunt trauma. Typical clinical findings include evidence of ongoing bleeding (up to and including hemorrhagic shock), with a large area of discoloration and/or bruising and many times a palpable mass. Use of anticoagulant medication is usually found during detailed anamnesis. A high index of suspicion and prompt directed therapy are required to have positive outcomes. The level of expertise required is not usually available outside of trauma centers in the rural areas, which adds to the raison d'être of trauma networks. More research is needed to clarify the optimal timing for reinitiating anticoagulation.

## Figures and Tables

**Table 1:** Activation criteria.

Criteria	Category I activation	Category II activation
Physiologic criteria	Blunt or penetrating trauma with unstable vital signs	Blunt trauma with stable vital signs Penetrating extremity trauma not meeting category I anatomic criteria
	Hemodynamic compromise as evidenced by (i) BP ≤ 90 systolic or (ii) PEDS ≤ 90 systolic	No hemodynamic compromise
	Respiratory compromise as evidenced by (i) Respiratory rate < 10 or >29 or (ii) RR < 20 in an infant < 1 year of age Needing ventilator assistance Altered mentation as evidenced by GCS ≤ 10, persistent unconsciousness or focal signs (i.e., posturing), posttraumatic seizures, or pupillary anomalies All patients (with or without sedation) requiring ET intubation Inability to intubate and anticipation of surgical airway Blood infusing to maintain vitals	No respiratory compromise GCS of 11, 12, or 13 Pregnancy ≤ 20 weeks (anything above is considered category I)
Anatomical criteria	Head and face (i) Open or depressed skull fractures (ii) Positive imaging for subdural hematoma, epidural hematoma, or subarachnoid bleed Chest & abdomen (iii) Chest trauma with instability/significant pain (i.e., flail chest and evidence of crush) (iv) Obvious signs of trauma (open pneumothorax, ongoing bleeding, extensive SQ emphysema) (v) Abdominal trauma with significant pain or obvious external signs (i.e., evisceration, open wounds, bleeding) (vi) Imaging evidence of tension pneumothorax or cardiac tamponade or a (+) E-FAST (vii) Signs of an unstable pelvis and/or bleeding Spinal cord (viii) Documented injury with sensory deficits or new-onset paralysis Extremities (ix) Crush or degloving injury (x) Pulselessness or evidence of impaired blood flow in the limb or ongoing bleeding (xi) Two or more proximal long bone fractures (xii) Traumatic amputation proximal to the wrist or the ankle Other injuries to include in category I trauma (i) Two or more body regions with potential life or limb threat (ii) Combination trauma with ≥20% TBSA burn (iii) Other injuries at the discretion of the ED MD	Blunt head trauma (except anticoagulation use: see below) History of blunt chest trauma with pain History of blunt abdominal trauma Blunt extremity trauma deemed significant but not meeting anatomic or physiologic criteria Penetrating extremity trauma not meeting anatomic or physiologic criteria
Mechanism of injury	Penetrating injury to the head, neck, chest, or abdomen with the suspicion of trauma to underlying structure or cavity and/with any of the physiologic criteria above Falls over 20 feet for adult patients Falls over 2 times the height or length of a pediatric patient MVC with any of the following: Prolonged extrication (≥20 min) (i) Rollover and/or ejection (partial or complete) (ii) Death in the same passenger compartment (iii) Intrusion into passenger compartment > 12 inches at the driver side or >18 inches at any other side (iv) Motorcycle with a rider thrown from the vehicle (v) Estimated speed above 20 MPH Auto vs. pedestrian with significant impact (damage to the vehicle) or >20 MPH or pedestrian ran over or thrown at any distance	Blunt injury to the head, neck, chest, or abdomen with the suspicion of trauma to the underlying structure or cavity without any of the physiologic criteria above Ground level or standing height falls while on any anticoagulation not meeting category I anatomic criteria All deceleration injuries not meeting anatomic or physiologic category I criteria MVC without significant impact (damage to the vehicle) or <20 MPH Auto vs. pedestrian without significant impact (damage to the vehicle) or <20 MPH
Transfer in patients	Any patient that is transferred from another facility for further definitive care meeting the above criteria	Any patient that is transferred from another facility for further definitive care meeting the above criteria
Additional criteria	At the discretion of the ED physician for patients that do not strictly meet the above criteria	At the discretion of the ED physician for patients that do not strictly meet the above criteria

Sources: (1) *Resources for Optimal Care of the Injured Patient 2014* (orange book)—ACS; (2) *Guidelines for Field Triage of Injured Patients*—CDC. BP: blood pressure; E-FAST: Focused Assessment with Sonography in Trauma with added limited echocardiography; ET: endotracheal; GCS: Glasgow Coma Scale; MVC: motor vehicle crash (including motorcycle crash); MPH: miles per hour of speed; SQ: subcutaneous.

## Data Availability

All the data collected for the work is depicted in the manuscript.
